# Evaluation of the Efficacy of Recent Caries Removal Techniques: An In Vitro Study

**DOI:** 10.7759/cureus.34432

**Published:** 2023-01-31

**Authors:** Nikhil Sharma, Suruchi Sisodia, Arvind Jain, Triveni Bhargava, Pratiksha Kumar, Kuldeep Singh Rana

**Affiliations:** 1 Department of Conservative Dentistry and Endodontics, Modern Dental College and Research Centre, Indore, IND; 2 Department of Conservative Dentistry and Endodontics, Government College of Dentistry, Indore, IND; 3 Department of Oral and Maxillofacial Surgery, Government College of Dentistry, Indore, IND; 4 Department of Oral Pathology and Microbiology, Government College of Dentistry, Indore, IND

**Keywords:** demineralized dentine, bur excavation, laser, papacarie, dental caries

## Abstract

Introduction: Dentistry is undergoing a gentle revolution that will consign drill and fill to history. In order to increase the acceptance of dental treatment, efforts are directed toward changing traditional painful dentistry into a new concept of painless dentistry. It is common practice to utilize burs for caries removal and cavity preparation. Chemomechanical caries removal is a painless procedure that uses a chemical substance to eradicate diseased dentine. With the Food and Drug Administration (FDA) approval of Erbium-doped yttrium-aluminum-garnet (Er: YAG) laser systems for caries removal and cavity preparation, the discipline of laser operational dentistry was born out of a desire to find a way to remove the decay without causing any pain or stress to the surrounding healthy tissue.

Aim and Objectives: This in vitro research aimed to assess the relative advantages of chemomechanical and laser caries extraction techniques in comparison to the more traditional bur technique. The efficacy of each method was evaluated by microscopic examination of samples treated with each experimental method. We also evaluated the efficiency of each method by recording the time required for caries excavation.

Material and Methods: The methods used for caries excavation were bur excavation, chemo-mechanical method, and laser method. Histological slices were produced after all the samples had been treated with the experimental techniques, and they were studied using a binocular light transmission microscope. The samples were then scored as ‘0’ for absence and ‘1’ for the presence of demineralized dentine. These scores and the time recorded for each method were subjected to statistical analysis.

Results: This study found no statistically significant difference in the effectiveness of the different approaches to removing caries; however, bur excavation was the quickest and chemo-mechanical was the slowest, with the latter not being useful in cases with low caries activity. The laser method of caries removal doesn’t remove caries existing in the undercut areas of the cavity thus making the use of bur mandatory.

Conclusion: With more practice and experience, the chemo-mechanical and laser methods can be used in a more efficient way to render painless operative procedures to patients.

## Introduction

The importance of preserving tooth structure, together with a patient-centered approach, is increasingly becoming the standard in dental practice. Tissue should be saved if feasible, intrusive procedures should be minimized, and artificial replacements for genuine tissues should be used only when necessary. Using minimally invasive restorative dental treatments shows respect for the healthy tissue and prevents further damage, extending the lifespan of the original tooth [1]. Due to the excessive tooth slicing anticipated to acquire opposition and restricted maintenance in ordinary pit planning, the concept of protection has grown much more popular as a result of constant advancements in the mechanical and adhesive qualities of dental filling materials.

The standard approach to pit design is based on Dr. G.V. Black's "Extension for Prevention" philosophy. This approach to dental caries management has been replaced by "Construction with Conservation" due to the development of new dental helpful materials, the rise of adhesive dentistry, and a superior understanding of the caries cycle and the tooth's true capacity for remineralization [2]. This concept involves finding caries early, assessing each patient's risk of developing caries, using various caries detecting agents, adopting a new careful methodology that involves more compact tooth configurations using new cavity designs and dental adhesives, and repairing the damage instead of replacing it. The goal is to preserve the natural tooth form. When it comes to the placement and replacement of reclamations, the mindset of minimally invasive dentistry is one that coordinates counteraction, remineralization, and negligible mediation. It includes the following different techniques: air abrasion [3], atraumatic restorative technique [4], sono abrasion [5,6], and chemomechanical caries method (CMCR) [7].

Drilling, no matter how carefully done, often removes healthy parts of the tooth as well as affects its durability in the long run. Furthermore, carrying out caries excavation procedures with burs becomes difficult in patients who are very apprehensive or have high levels of anxiety. To address these issues, chemo-mechanical eradication of caries was developed as an alternative to conventional caries treatment. Chemo-mechanical caries removal is a non-invasive technique for removing damaged dentine. With this approach, not only are infected areas of the tooth removed but the pulp is also shielded from unnecessary discomfort and irritation. Instead of amalgam, which requires manually cutting a cavity to sustain the repair, composite resins or glass ionomers, which attach to the dentine surface, are appropriate for repairing the cavities created by this procedure.

After recognizing the shortcomings of CariSolv®, the new reagent Papacarie® was developed. In particular, it is mostly composed of papain, chloramine, and toluidine blue. In its natural form, papain is an enzyme found in the latex of mature *Carica papaya* plants, namely in their green papaya fruit and leaves. It is structurally linked to human pepsin and functions as a debriding, anti-inflammatory, and antibacterial endoprotein. Papacarie is an antimicrobial, biocompatible gel that speeds healing, reduces the need to remove good tissue, and enhances tissue preservation. When using the gel, no smear layer is seen to form. Various amounts of Papacarie (2%, 4%, 6%, 8%, and 10%) were tested for cytotoxicity in cultured fibroblasts in vitro. It was determined that Papacarie was non-cytotoxic in vitro fibroblast culture and was biocompatible with oral tissues; thus, it could be used safely [8].

Another method of caries removal, as listed above, is by the use of lasers. The noise level and vibration level of the laser are both low compared to those of a rotary bur. It is said that the need for local anesthesia may be avoided since laser therapy is so painless. Research on the effects of neodymium (Nd) and carbon dioxide (CO_2_) lasers on tooth hard tissues was conducted in the 1970s. Initial research on the effects of CO_2_ lasers on teeth revealed significant structural damage, carbonization, fissuring, and increased mineralization as a result of the removal of organic substances [9]. Loss of the odontoblastic layer due to CO_2_ laser usage was also mentioned as a drawback. However, recent developments in laser technology have uncovered biocompatible interactions. Dental hard tissues were used to evaluate the Erbium-doped yttrium-aluminum-garnet (Er: YAG) laser's capacity to ablate (or evaporate). Scaling, minor surgery on soft tissues, and the excision of cavities have all been accomplished with this laser.

This in vitro study aimed to compare the efficiency of three techniques for removing caries: the round bur, Papacarie Duo^TM^ gel, and the Er:YAG laser. This study aimed to determine if the Papacarie or the Er:YAG laser was more effective for removing carious tooth tissue and, if so, by how much shorter a time period.

## Materials and methods

The study was conducted with the ethical clearance of Bharati Vidyapeeth Dental College and Hospital, Pune, Maharastra, India (Approval number: BVDCH/2020/14). The study was conducted at the dental clinic of Bharati Vidyapeeth Deemed University, Pune, India, and included staff from the departments of oral pathology, microbiology, oral biology, and histology. Thirty human molars that had to be extracted due to periodontal disease were collected from the exodontia department at Bharati Vidyapeeth Dental College and Hospital, Pune, for the purpose of this study. After the teeth were extracted, ultrasonic scalers were used to remove any remaining soft tissue or lingering debris. Each tooth was given a thorough cleaning using a pumice and water slurry to get rid of debris, then rinsed with distilled water and dried for five seconds using compressed air. Exclusion criteria include grossly carious teeth with no remaining coronal structure, teeth with caries extending into the enamel only, and teeth with previous fillings. Inclusion criteria include teeth with carious crowns.

Procedure

After extraction, teeth were preserved in 10% formalin. Thirty cavitated teeth were arbitrarily split into three sets of 10 teeth each. The following caries-removal protocols were used for each group:

(i) Group A: Using a slow-spinning diamond disk, we sectioned the teeth through the middle of the lesions in the mesiodistal longitudinal plane. Each tooth had one-half of its surface treated with a round bur and a high-speed air rotor handpiece (sub-group A1), while the other half was treated with Papacarie Duo^TM^ gel (sub-group A2) to get rid of caries. For sub-group A1, we used intermittent cutting until we could tell that all of caries had been removed (by sight and feel). When treating a carious lesion with sub-group A2, the gel was applied, and the area was left undisturbed for 30 seconds. A spoon excavator was used to carefully scrape away hazy gel without exerting any force, and then the new gel was poured into the excavation site. Carious dentin was removed until the gel was clear. The cavity was then cleaned with a damp cotton pellet after the gel was taken out. The time taken to treat each sample was recorded using a stopwatch. Thus, we obtained 20 samples in Group A to compare the efficacy of round burs and Papacarie Duo.

(ii) Group B: The same procedures were used as Group A to collect the samples. Caries was removed from one side of each tooth in this sample using a round bur and high-velocity air rotor handpiece (sub-group B1) and from the opposite side using an Er: YAG laser (sub-group B2). A round bur was used in a similar fashion on sub-group B1 samples as it was on sub-group A1 samples. Emission was at 2.94 m, pulse energy was 200 MJ, and repetition rate was 20 Hz to get 4 watts from the laser system. It was decided to use a non-contact technique for caries removal. Handpiece R14's water spray kept the laser point a constant 1 mm from the tooth's surface while it was held there. It was constantly moved across the surface for wide cutting, and it was focused on the spot where caries appeared deep for deep cutting. A laser beam was used on the sample until all the caries were excavated, as determined by visual and tactile sensation. The time taken to treat each sample was recorded using a stopwatch. Thus, even in Group B, we obtained 20 samples to compare the efficacy of round burst and Er: YAG laser.

(iii) Group C: The samples in this group followed the same procedures as those in Groups A and B. In this group, teeth with caries were treated by applying Papacarie Duo gel (sub-group C1) to one half of each tooth and then using an Er: YAG laser (sub-group C2) on the other half. Comparable procedures were carried out on samples in sub-group C1 and samples in sub-group C2 to replicate the results obtained in subgroups A2 and B2, respectively. The time taken to treat each sample was recorded using a stopwatch. Thus, we obtained 20 samples in Group C to compare the efficacy of Papacarie Duo and Er: YAG lasers. All the samples were thoroughly rinsed and stored in 10% formalin until further study.

Evaluation of caries excavation

Tactile and visual methods of caries detection revealed the full excavation of all caries in all samples. A keen explorer was utilized to probe the dentinal wall. In cases where the explorer was able to be withdrawn from the tooth without encountering any resistance, it was determined that all caries had been successfully eliminated. And therefore, excavation was done until the entire dentinal surface was hard and glossy.

Preparation of treated samples for microscopic examination

Before being examined under a microscope, the treated samples were decalcified in 10% nitric acid corrosive for five days at room temperature. After washing, the samples were dried in increasing concentrations of isopropyl alcohol (IPA), then cleaned in xylene, and finally embedded in paraffin. The existence of demineralized dentine (resembling caries) was analyzed by cutting 5 mm segments in succession, staining them with hematoxylin and eosin, and then photographing the results under a microscope. The histological sections of each group were scored as 0 for the absence of demineralized dentin and 1 for the presence of demineralized dentine, and the scoring was subjected to statistical analysis using the Z-test. The mean time in seconds to remove caries from all the samples of each group was calculated and analyzed statistically to evaluate the efficiency of experimental techniques using the Wilcoxon test.

## Results

This in vitro study set out to evaluate the relative success of many caries eradication strategies. The methods used for caries excavation were bur excavation, chemo-mechanical method, and laser method. The efficacy of the three methods was obtained by histological examination of the treated samples. For efficiency, the time taken for caries excavation was recorded. The histological sections were scored as ‘0’ for absence and ‘1’ for the presence of demineralized dentine. These scores and the time recorded for each method were subjected to statistical analysis. 

The total number of samples with caries in sub-group A1 is one, and the total number of samples with caries in sub-group A2 is three. The result is not significant at p <0.05. The proportion of responses for Observation 1 is 0.1. The proportion for Observation 2 is 0.3 (Table 1).

**Table 1:** Evaluation of efficacy: Group A (Bur vs. Papacarie) '0’ for absence of demineralized dentine; ‘1’ for the presence of demineralized dentine

Sample No. (Group A1)	Score	Sample No. (Group A2)	Score
A1-1	0	A2-1	0
A1-2	0	A2-2	0
A1-3	0	A2-3	1
A1-4	0	A2-4	0
A1-5	0	A2-5	0
A1-6	1	A2-6	1
A1-7	0	A2-7	0
A1-8	0	A2-8	0
A1-9	0	A2-9	0
A1-10	0	A2-10	1

There was one sample with caries in sub-group B1 and two samples with caries in sub-group B2. The result is not significant at p <0.05. The proportion of responses for Observation 1 is 0.1. The proportion for Observation 2 is 0.2 (Table 2).

**Table 2:** Evaluation of efficacy: Group B (Bur vs. Laser) ‘0’ for absence of demineralized dentine; ‘1’ for the presence of demineralized dentine

Sample No. (Group B1)	Score	Sample No. (Group B2)	Score
B1-1	0	B2-1	1
B1-2	0	B2-2	0
B1-3	0	B2-3	0
B1-4	0	B2-4	0
B1-5	0	B2-5	0
B1-6	0	B2-6	0
B1-7	0	B2-7	0
B1-8	1	B2-8	1
B1-9	0	B2-9	0
B1-10	0	B2-10	0

There were four samples with caries in sub-group C1 and one sample with caries in sub-group C2. The result is not significant at p <0.05. The proportion of responses for Observation 1 is 0.4. The proportion for Observation 2 is 0.1 (Table 3).

**Table 3:** Evaluation of efficacy: Group C (Papacarie vs. Laser) ‘0’ for absence of demineralized dentine; ‘1’ for the presence of demineralized dentine

Sample No. (Group C1)	Score	Sample No. (Group C2)	Score
C1-1	1	C2-1	0
C1-2	0	C2-2	0
C1-3	1	C2-3	0
C1-4	0	C2-4	0
C1-5	1	C2-5	1
C1-6	0	C2-6	0
C1-7	1	C2-7	0
C1-8	0	C2-8	0
C1-9	0	C2-9	0
C1-10	0	C2-10	0

Time taken by Papacarie is significantly higher than round bur (p<0.05 by Wilcoxon Test) as shown in Tables 4-5.

**Table 4:** Evaluation of Efficiency: Group A (Bur vs Papacarie)

Sample No.	Time (Sec.)	Sample No.	Time (Sec.)
A1-1	127	A2-1	300
A1-2	59	A2-2	180
A1-3	121	A2-3	270
A1-4	53	A2-4	210
A1-5	63	A2-5	210
A1-6	126	A2-6	300
A1-7	66	A2-7	210
A1-8	74	A2-8	240
A1-9	117	A2-9	270
A1-10	125	A2-10	270

**Table 5:** Evaluation of mean efficiency: Group A (Bur vs Papacarie)

		Mean	N	Standard Deviation	Standard Error Mean	p-value
Group A	Bur	93.1000	10	32.27469	10.20615	0.005
	Papacarie	246.0000	10	41.95235	13.26650	

Time taken by Er:YAG laser is significantly higher than bur (p<0.05 by Wilcoxon Test) as shown in Tables 6-7.

**Table 6:** Evaluation of Efficiency: Group B (Bur vs Laser)

Sample No.	Time (Sec.)	Sample No.	Time (Sec.)
B1-1	70	B2-1	144
B1-2	127	B2-2	212
B1-3	64	B2-3	133
B1-4	128	B2-4	210
B1-5	59	B2-5	134
B1-6	48	B2-6	97
B1-7	121	B2-7	193
B1-8	67	B2-8	129
B1-9	109	B2-9	187
B1-10	93	B2-10	166

**Table 7:** Evaluation of mean efficiency: Group B (Bur vs Laser)

		Mean	N	Standard Deviation	Standard Error Mean	p-value
Group-B	Bur	88.6000	10	30.64927	9.69215	0.005
	Laser	160.5000	10	38.93941	12.31372	

Time taken by Papacarie is significantly higher than laser (p<0.05 by Wilcoxon Test) as shown in Tables 8-9.

**Table 8:** Evaluation of Efficiency: Group C (Papacarie vs Laser)

Sample No.	Time (Sec.)	Sample No.	Time (Sec.)
C1-1	300	C2-1	194
C1-2	150	C2-2	80
C1-3	240	C2-3	163
C1-4	240	C2-4	155
C1-5	180	C2-5	86
C1-6	180	C2-6	108
C1-7	210	C2-7	116
C1-8	300	C2-8	197
C1-9	210	C2-9	128
C1-10	240	C2-10	149

**Table 9:** Evaluation of mean efficiency: Group C (Papacarie vs Laser)

		Mean	N	Standard Deviation	Standard Error Mean	p-value
Group C	Papacarie	225.0000	10	49.49747	15.65248	0.005
	Laser	137.6000	10	41.09934	12.99675	

When the average time taken by each experimental method for all the samples treated was calculated, the following values were obtained: Round bur with air-rotor took 90.85 seconds, Papacarie Duo gel took 235.5 seconds, and Er: YAG laser took 149.05 seconds. 

## Discussion

Among all chronic diseases, dental caries has the largest worldwide prevalence. Loss of function and aesthetics, in addition to increased pain and suffering, has a negative impact on a person's health and well-being. Caries removal is frequently avoided due to the stigma associated with it. Developing novel strategies for caries elimination as an alternative to the status quo has long been a focus of dental science. Research in this area has led to the development of new tools for preparing cavities for the placement of advanced adhesive restoratives, which is important for a number of reasons (including speed, painless preparation, cost-effectiveness, clinical application, safety, and correct, conservative cavity preparation). For the better part of the 20th century, dentists relied on a system of categorization developed by Dr. G.V. Black, which led to treatments like root canal therapy and crown lengthening for carious lesions. As a result, the concept of using an extension to combat crime became popular.

In the 1970s, an endodontist named M. Goldman came up with the concept of chemo-mechanical caries eradication while cleaning the root canals with 5% sodium hypochlorite (5% NaOCl) [10]. The idea of chemically removing caries came about because this chemical could destroy carious dentin. The chemomechanical method of caries eradication was first published in 1975 by Habib et al. using 5% sodium hypochlorite (NaOCl) and then refined in 1976 by Goldman et al. using GK-101 [11]. This solution of 0.05% N-monochloroglycine was made by diluting NaOCl with water and buffering it with sodium hydroxide, sodium chloride, and glycine (NMG). In 1984, N-monochrome-2-aminobutyrate (NMAB), also known as GK-101E (Caridex), was released. Although Caridex was effective in chemical caries removal, it was replaced in 1997 by the Carisolv system, developed by Medi Team, Sweden, due to its unpleasant flavor, lengthy process time (10-15 minutes), usage of huge quantities of solution (200-500 ml), challenging delivery system, and high costs [10].

In 2003, a new reagent was created in Brazil as an answer to the existing reagent's limitations (such as its short shelf life, high corrosiveness, demand for specialist equipment, and high cost). Papain gel, often known as Papacarie, was initially launched as a chemomechanical caries remover by Formula and Acao in Sao Paulo [12]. In 1997, the Er: YAG laser system for caries removal and cavity preparation was approved by the Food and Drug Administration (FDA), marking the beginning of the field of laser-operative dentistry and its mission to find a way to remove decay without causing pain or trauma to the patient or the surrounding healthy tissue. Enamel, dentin, and decay are all destroyed by the Er: YAG laser pulse's violent contact with water molecules on the tooth tissue surfaces. When the interstitial water trapped inside the mineral substrate expands rapidly below ground, it creates a massive increase in volume, which in turn causes the surrounding material to be blasted away. Lasers used in dentistry work by converting their radiant energy into thermal energy. As the temperature rises, the denatured tissue may vaporize and carbonize, or it may melt and then recrystallize in the case of hard tissue [13]. All three experimental methods of caries excavation included in this study claim to be quick and remove all the carious tissue from the teeth. We conducted the study to check, in vitro, the efficacy and efficiency of these methods. Since bur excavation is the most commonly used method of caries excavation worldwide, we compared the chemo-mechanical and laser methods with it.

In 2011, a new iteration of the product called Papacarie Duo was developed, and it retains the product's original effectiveness while also including a number of desirable qualities, such as a longer shelf life and the elimination of the refrigeration requirement. The higher viscosity of the gel also facilitates pinpoint placement with little overspray [14]. As per the information gathered through journals and the internet, only one study has been conducted to check the performance of Papacarie Duo since its inception. So it was included in our study. When it comes to removing caries, laser technology is another option that does not involve any discomfort. Caries is often removed using the Er: YAG laser. Since it is readily absorbed by both water and hydroxyapatite, this mid-infrared radiation (emitting at a wavelength of 2.94 m) may one day be used to treat hard tissues. According to Craig B. Gimbel's recommendations, we used an Er: YAG laser to excavate cavities from sample teeth [9].

Visual and tactile sensation criteria are more reliable for caries detection. According to Dennison and Hamilton, the most reliable clinical method for assessing the severity of a carious lesion is to examine the exposed area for differences in surface texture [15]. After caries excavation from all the samples, the samples were prepared for histological examination. The histological examination was done to evaluate the presence or absence of demineralized dentine in each sample, which in turn indicated the efficacy of that particular method. The teeth before and after treatment were stored in 10% formalin till further examination, as it helps in tissue fixation. Decalcification was done by placing the samples in 10% nitric acid for five days, which softens the hard structure, to facilitate perfect sectioning later. Following decalcification, the samples were dehydrated in increasing concentrations of IPA, cleared in xylene, and embedded in wax to harden the tissue and hold the sample steady while sectioning in the microtome. These sectioned samples were then stained with hematoxylin and eosin, which is the most commonly used stain for studying dentine structure.

The findings of this research show that the round bur with the air-rotor technique of caries extraction is the most effective. The result was a dull, firm, and shiny dentinal surface. The findings of this study are consistent with the hypotheses of Jawa et al. [2] and Neves et al. [16], that the air rotor was the most effective tool for eliminating caries due to its tendency to excessively prepare the cavities due to its insensitivity to touch input. When even perfectly sound tooth structure could be removed due to lack of resistance, there were minimal chances of affected discolored dentin being preserved. This resulted in an absolutely clear, hard, and glossy surface post-treatment and so only two out of the 20 samples treated with round bur showed remaining caries when histologically examined. There was no statistically significant difference in effectiveness between Papacarie Duo gel and the other two experimental techniques, despite the fact that it failed to eradicate caries in seven of the 20 samples. One possible explanation for this result is that using Papacarie requires less thorough preparation than using a mechanical drill. This bacterium may be discovered in otherwise healthy dentin, which is why the chemo-mechanical approach is used to maintain it. The findings of Jawa et al. are consistent with ours. Papacarie Duo gel, like CariSolv, promises to selectively eradicate caries, but it does so in a different way. Papain, chloramines, toluidine blue dye, water, salts, and thickeners are their main components. Its primary component is the proteolytic enzyme papain. It kills bacteria, prevents further growth of bacteria, and reduces inflammation. Another reason for the remaining bacteria, as suggested by Yazici et al., likely has something to do with the smear layer [17]. CariSolv, which has been found to reduce or eliminate the smear, was the subject of the research.

Although there was no statistically significant difference between the three methods, this study found that the Er: YAG laser was more effective than the bur excavation approach and the Papacarie Duo gel. Nair et al., in their investigation, reported that Er: YAG irradiation produces clean, sharp margins in enamel and dentin [18]. Shigetami et al. compared Er: YAG laser caries removal to standard air-rotor treatment and found no statistically significant difference in efficacy between the two [19]. The findings from the current investigation are consistent with these hypotheses. According to the results of this investigation, in terms of speed, the air rotor was the most effective method, the Er: YAG laser was second, and the Papacarie Duo gel was last. Using visual and tactile sensation criteria, there was a statistically significant difference in the mean time needed to dig completely. Similar results were found in in vivo studies done by Matsumoto et al. [14]. Multiple studies have shown that Papacarie gel is much less effective in removing caries than air rotor. Although Lozano-Chourio et al. suggested that age, cavity severity, tooth type, and cavity size might have contributed to the time disparities, it remains unclear why the times varied so widely [20-22].

The longer time required for caries excavation using the chemo-mechanical approach compared to the mechanical (air rotor) method in this study may be attributable to some of the aforementioned factors. According to the study by Yamada et al., the air rotor was the quickest, followed by the laser, and the Carisolv was the slowest [23]. Our results are in confirmation with that study as far as the laser and bur parameters are concerned. More time required for the laser method compared to round bur with air rotor excavation could also be attributed to the operator’s inexperience with this technique, although preliminary training was done prior to the commencement of the study. Irrespective of the technique used for caries excavation, the main goal of the complete elimination of the diseased tissue should be achieved.

## Conclusions

Lasers have been able to non-invasively treat various lesions of the oral cavity. With more practice and experience, the chemo-mechanical and laser methods can be used more efficiently to provide patients with painless operative procedures. Irrespective of the method of caries removal used, the ultimate goal is to remove all the diseased portions of the tooth with less cutting and less removal of healthy tissue, leaving natural, healthy tissue as solid and as strong as possible.
